# Editorial: From interventional pearls to pioneering technologies in transcatheter treatment of congenital heart defects

**DOI:** 10.3389/fcvm.2024.1484393

**Published:** 2024-09-10

**Authors:** Raymond N. Haddad, Damien Kenny, Francesco Sturla, Gianfranco Butera

**Affiliations:** ^1^ M3C-Necker, Hôpital Universitaire Necker-Enfants Malades, Assistance Publique-Hôpitaux de Paris (AP-HP), Paris, France; ^2^ Paediatric Cardiology, Children’s Health Ireland, Dublin, Ireland; ^3^ 3D and Computer Simulation Laboratory, IRCCS Policlinico San Donato, Milan, Italy; ^4^ Department of Electronics, Information and Bioengineering, Politecnico di Milano, Milan, Italy; ^5^ Department of Interventional Cardiology, Bambino Gesù Children’s Hospital, IRCCS, Rome, Italy

**Keywords:** absorbable metal stents, atrial flow regulator, holography-guided procedural planning, multimodality image fusion, pulmonary valve implantation, pulmonary flow restrictors, 3D imaging techniques

**Editorial on the Research Topic**
From interventional pearls to pioneering technologies in transcatheter treatment of congenital heart defects

## Introduction

In the rapidly evolving field of congenital and pediatric cardiology, we stand at the threshold of transformative advancements. Over recent decades, groundbreaking progress in transcatheter interventions and the integration of sophisticated 3D technologies have revolutionized the treatment of congenital heart defects (CHDs). Our latest Research Topic, “From Interventional Pearls to Pioneering Technologies in Transcatheter Treatment of CHDs,” delves into this innovative landscape, exploring the most recent breakthroughs and practical insights from the forefront of pediatric interventional cardiology.

This collection, featuring contributions from world-renowned experts and emerging thought leaders, highlights a wide spectrum of new devices, advanced imaging techniques, and innovative procedural strategies. From the potential and challenges of absorbable metal stents (AMS) to the precision of holography-guided valve implantation, and from the benefits of multimodality image fusion to novel approaches in managing complex CHDs, these 21 insightful manuscripts provide a comprehensive view of contemporary advancements. Each study brings a unique perspective, whether by pioneering new devices, optimizing existing technologies, or refining procedural techniques.

The insights presented here are poised to significantly enhance clinical practice and patient outcomes, making it crucial for the interventional pediatric cardiology community to stay informed of these developments. As we explore these pioneering technologies and interventional pearls, this editorial seeks to highlight the essential updates and emerging trends that are reshaping the treatment paradigms for CHDs. Join us in navigating the forefront of innovation, as we illuminate the path toward optimal clinical and procedural outcomes in this ever-dynamic field.

## Advances and challenges in pediatric cardiac stenting and occlusion devices

### AMS in pediatric cardiology

In their review, McLennan et al. explore the development and clinical application of AMS in pediatric cardiology, particularly for CHD. Although AMS technology has advanced, it has yet to achieve the desired degradation benchmarks, with current commercial devices underperforming in long-term applications. The review examines various materials used in AMS, such as magnesium, zinc, and iron, noting their differing mechanical strengths and degradation rates. Despite the potential benefits of AMS, significant challenges remain in material design and clinical application. The review emphasizes ongoing efforts to refine AMS technology for improved treatment of vascular anomalies in pediatric patients.

### Bilateral asymmetric single-rivet occluder for patent foramen ovale (PFO) closure with reserved interatrial septal puncture area

Wei et al. successfully created a PFO model in 6 pigs using high-pressure balloon dilation, achieving an average unclosed PFO diameter of 3.56 ± 0.25 mm. They then employed a novel bilateral asymmetric single-rivet occluder to close the PFO, resulting in complete closure in all pigs without residual shunting, as verified by transesophageal echocardiography. After 3 months, the occluder facilitated successful interatrial septal puncture procedures through a designated area. Histological analysis post-euthanasia showed intact devices with endothelial coverage and no nitinol wire fractures. The device demonstrated excellent closure efficacy, biocompatibility, and puncturability, indicating its promising potential for future catheter-based interventions on the interatrial septum.

### Occlutech atrial flow regulator (AFR) for CHD

Butera et al. evaluated the Occlutech AFR in 40 patients with CHD, pulmonary hypertension (PH), or cardiomyopathy, including children. The median age at implantation was 58.5 months, and the median weight was 17 kg. The implantation success rate was 100%, though three cases of device thrombosis were noted. Two patients with dilated cardiomyopathy on ECMO died during hospitalization. At a median follow-up of 330 days, 92.5% of patients were alive, with 20 showing improvement in the NYHA class. The study suggests that AFR implantation is a promising short-term treatment for CHD, severe PH, and cardiomyopathy in children.

### Left pulmonary artery (LPA) stenting and its impact on lung development

Callegari et al. evaluated the effects of LPA stenting on bronchial size, pulmonary volumes, and lung function in 49 patients with single ventricle physiology, including 17 who received LPA stents. The study found that patients with LPA stents had a significantly larger right-to-left bronchus area ratio (*p* < 0.001) but also showed a trend toward deformation of the left main bronchus (LMB) and reduced left lung volume. Early stent placement was linked to smaller LMB size, slightly smaller left lung volumes, and a higher rate of abnormal spirometry results. These findings suggest that early LPA stenting might adversely affect lung development, although it is uncertain whether these effects are due to the stent or pre-existing conditions.

### Coarctation (CoA) stenting in very low birth weight infants

Mini et al. demonstrated the feasibility of CoA stenting in very low birth weight (VLWB) and extremely low birth weight (ELWB) preterm infants. The study included successful interventions in three infants with weights of 1,350 g, 1,200 g, and 600 g. Performed between 2020 and 2022, the procedures used the femoral artery for access and were guided by transthoracic echocardiography without the use of contrast agents, which was particularly advantageous for patients with renal failure. All interventions were free of complications, with preserved left ventricular function and no need for stent re-intervention. Two patients had their stents removed successfully after 73 and 110 days, respectively, while the third is pending further surgery. This technique provides a safe and effective bridge to future surgical interventions in VLWB and ELWB infants.

### Right ventricular outflow tract (RVOT) stenting in Fallot physiology

Prakoso et al. evaluated the efficacy of RVOT stenting in 32 patients with unrepaired Fallot physiology, including 10 adults and 22 children. Post-procedure, there was a significant improvement in patients’ oxygen saturation, rising from 58.56% to 91.03% (*p* < 0.001), and left ventricular ejection fraction, which increased from 64.00% to 75.09% (*p* = 0.001). Adults received longer stents (43.60 mm) compared to children (31.77 mm). The median follow-up period was 13.5 months, with the median time to total repair being 3 months. The study concluded that RVOT stenting is both safe and effective, facilitating somatic growth and pulmonary artery rehabilitation before total repair, with no reported intraprocedural deaths.

## Innovative technologies and techniques in cardiac interventions

### Holography-guided Venus *P*-valve implantation

D’Aiello et al. present a case series exploring the use of holography-guided procedural planning for Venus P-valve implantation in patients with LPA stents. Among 17 patients scheduled for the procedure between January and October 2023, 16 (94%) successfully underwent implantation, including three with LPA stents. The integration of 3D holographic models, based on pre-operative CT scans, allowed for precise procedural planning, achieving a 100% success rate with no complications. Follow-up results showed favorable hemodynamic outcomes in patients aged 20-63. This study underscores the effectiveness of employing advanced imaging techniques, such as 3D holography, in managing anatomically complex cases with Venus P-valve implantation.

### 3D-printed models and custom-made mock loops for optimizing percutaneous pulmonary valve implantation (PPVI)

Odemis et al. investigated the use of custom-modified cardiovascular mock loops with patient-specific 3D-printed pulmonary artery models to refine percutaneous PPVI for patients with severe pulmonary regurgitation following Fallot repair. They conducted 39 experiments using five different patient geometries and valve sizes ranging from 26 to 32 mm, across cardiac outputs of 3, 4, and 5 L/min. The study found that the pressure gradients and regurgitation fractions of the tested valves were closely aligned with those observed in actual procedures. Notably, different valve sizes exhibited improved hemodynamic performance in two patients compared to the valves used in their real procedures, underscoring the potential of this approach to enhance PPVI outcomes.

### Multimodality image fusion in cardiac catheterization

Buytaert et al. compared conventional biplane angiography with multimodality image fusion using 2D-3D registration (MMIF2D−3D) during cardiac catheterizations for CHD patients. The study found that MMIF2D−3D significantly reduced lateral plane fluoroscopy time by 69.6%, which led to notable reductions in radiation exposure: a 43.9% decrease in air kerma normalized by body weight and a 39.3% decrease in dose area product normalized by body weight. Although there was a reduction in contrast volume normalized by body weight, this change was not statistically significant.

### Bovine pulmonary visceral pleura in bioprosthetic heart valves

Lu et al. investigated bovine pulmonary visceral pleura (PVP) as a potential material for bioprosthetic heart valve cusps. PVP valves, treated with 0.625% glutaraldehyde and mounted on nitinol stents, underwent rigorous mechanical testing, simulating 100 million cycles, and were then implanted in six pigs. The valves showed no considerable damage or tears during testing. *In vivo*, they performed well, with open cusps, and no signs of thrombosis, calcification, inflammation, or fibrosis were observed over four months. Histological analysis confirmed the preservation of elastin and collagen fibers in the PVP, with no calcific deposits. These results indicate that PVP bioprosthetic valves demonstrate strong mechanical durability and biocompatibility, suggesting their potential for long-term use.

### Microvascular plugs (MVPs) as pulmonary flow restrictors (PFRs)

Haddad et al. investigated the use of MVPs as endoluminal PFRs for CHDs in infants. The study involved implanting 28 PFRs (7 MVP-5Q, 12 MVP-7Q, 9 MVP-9Q) in 14 patients with a median age of 1.6 months and a median weight of 3.1 kg. The procedure resulted in a significant reduction in oxygen saturation and Qp/Qs ratio. Postoperative recovery was generally favorable, with all patients discharged from the ICU after a median of 3.5 days. Out of the 28 PFRs, 14 (50%) were successfully explanted after a median of 4.3 months. However, there were three fatalities due to non-cardiac causes. Overall, PFRs demonstrated effectiveness in managing pulmonary over-circulation in CHDs with positive outcomes and low complication rates.

### Fast atrial sheath traction (FAST) technique for atrial septal defect (ASD) closure

Haddad et al. assessed the effectiveness of the FAST technique for transcatheter closure of ASDs in 17 patients, predominantly male (64.7%), with a median age of 9.8 years and a median weight of 34 kg. This novel approach was utilized for ASDs with absent aortic rims or ASD size-to-body weight ratios greater than 0.9. The FAST technique involved a median ASD size of 19 mm and a device size of 22 mm. The procedure was performed with a median fluoroscopy time of 4.1 min and resulted in no complications. At 13 months of follow-up, all patients exhibited complete clinical recovery and full shunt closure, indicating that the FAST technique enhances procedural outcomes by simplifying implantation and minimizing risks.

## Case studies and unique approaches in cardiac procedures

### Transcatheter intervention for Fontan circulation complications

Schaffner et al. describe a novel transcatheter intervention for a 39-month-old with failing Fontan circulation and plastic bronchitis. The procedure modifies the traditional innominate vein turn-down method by placing a covered stent (Viabahn 7/19 mm) at the proximal end of a modified fenestration, linking the innominate vein to the right atrial appendage, and using an Amplatzer Duct Occluder (ADO) I 10/8 mm to close the distal innominate vein and relieve thoracic duct (TD) pressure. While not originally designed for TD decompression, this approach provides a safer, non-surgical alternative. Despite initial complications that required emergency resuscitation, notable hemodynamic and respiratory improvements were achieved. Long-term follow-up is necessary to confirm its effectiveness and durability.

### Complex device retrieval techniques

Xiang et al. detail the challenging retrieval of an embolized Amplatzer septal occluder from the descending thoracic aortic isthmus. Although the percutaneous retrieval using an Amplatz Goose Neck snare was successful, the procedure faced significant hurdles. The device's angulation in the aorta complicated capture, requiring precise maneuvering. Additional challenges included the need for a larger replacement device and unexpected intraoperative cardiac arrest. These difficulties highlight the need for advanced skills and meticulous planning in percutaneous retrieval techniques to manage complex device migrations and minimize risks.

### Thoracic endovascular aortic repair (TEVAR) for complex patent ductus arteriosus (PDA) closure

Jenab et al. introduce an innovative method for managing large, complex PDA in adults using TEVAR with a non-touch exclusion technique. They treated a 27-year-old male with a 20.2 mm PDA and severe pulmonary symptoms using a custom Zenith Alpha Thoracic Endovascular Graft. The procedure, performed under conscious sedation, involved deploying the graft in zone 2, which covered the left subclavian artery and achieved complete PDA closure. Post-surgical imaging confirmed no endoleaks and successful PDA occlusion. This technique offers a promising, less invasive alternative to traditional PDA closure methods, particularly for high-risk patients with complex anatomies.

## Comparative outcomes and long-term results in pediatric cardiac interventions

### Comparison of percutaneous vs. perventricular device closures for perimembranous ventricular septal defects (PmVSDs)

Huang et al. compared percutaneous (PCP) and perventricular (PVP) ultrasound-guided device closures for PmVSD in 205 patients. Both techniques showed comparable success rates (PCP 88.4%, PVP 92.5% intention-to-treat; PCP 88.4%, PVP 89.3% as-treated), with 5 of 8 percutaneous failures successfully managed by shifting to perventricular closure. PCP patients had a smaller median defect diameter (4 mm vs. 5.2 mm in PVP) and experienced significantly shorter ventilation time, drainage volume, and hospital stay (all *p* < 0.001). PCP had no severe adverse events, while PVP had a 3.0% rate of such events. The study suggests that PCP may offer quicker recovery and could be preferred for certain patients.

### ADO II vs. Lifetech Konar-MF VSD occluder (MFO) for PmVSD closure

Haddad et al. and Yildiz et al. investigated the outcomes of ADOII and MFO for pmVSD closures in children. Haddad et al. studied 77 children (mean age 3.7 ± 3.1 years, mean weight 13.3 ± 7.1 kg), finding a 100% implantation success rate with ADOII (used in 44 patients) vs. 90.9% with MFO (used in 33 patients). MFO was associated with higher complication rates, including two embolizations and one grade-2 aortic regurgitation. At 24 months, freedom from residual shunt was 90.62% for MFO and 89.61% for ADOII. Conversely, Yildiz et al. focused on 52 children under 10 kg, comparing outcomes with ADOII (22 children, median age 11 months, weight 7.4 kg) and MFO (30 children, median age 11 months, weight 8 kg). ADOII was used for smaller defects (left ventricular diameter 4.6 mm, right ventricular diameter 3.5 mm), while MFO addressed larger defects (left ventricular diameter 7 mm, right ventricular diameter 5 mm). MFO demonstrated shorter procedural and fluoroscopy times (*p* < 0.05) and showed advantages in handling larger defects and smaller subaortic rims. Both studies indicate that ADOII is better for smaller defects with fewer complications, while MFO is preferable for larger defects despite its higher complication rate.

### Risk factors for heart block post-PmVSD closure

Jiang et al. analyzed 1,076 pediatric patients who underwent transcatheter device closure for pmVSD between June 2002 and June 2020. Postprocedural heart block occurred in 234 (21.8%) patients, with right bundle branch block being the most common (74.8%), followed by left bundle branch block (16.2%) and atrioventricular block (5.6%). Of the five patients with complete atrioventricular block, three required permanent pacemakers, one recovered normal rhythm, and one experienced sudden cardiac death. Most heart blocks (97.9%) developed within a week. Risk factors included thin-waist occluders (OR: 1.759) and oversized devices (OR: 1.809). However, positioning the left disk within aneurysmal tissue reduced the risk (OR: 0.568). Notably, 138 patients returned to normal cardiac conduction.

### Midterm outcomes of transcatheter PDA closure with Amplatzer devices

Bruckheimer et al. conducted a retrospective review of 762 patients (median age 2.6 years, median weight 13 kg) who underwent transcatheter PDA closure with Amplatzer devices from January 2008 to April 2022. Overall success was achieved in 758 patients (99.5%). The device distribution included 296 (38.8%) ADOII, 418 (54.8%) Piccolo, and 44 (5.8%) Amplatzer vascular plug (AVP) II. The ADOII group had smaller patients (median weight 15.8 kg) with larger PDA diameters (2.3 mm) compared to the Piccolo group (20.5 kg, 1.9 mm). Closure rates at 6 months were similar across devices (ADOII 99.6%, Piccolo 99.7%, AVPII 100%). Four embolizations occurred (two ADOII, two Piccolo), with minor stenosis rates of 1% for ADOII and 0.2% for Piccolo. Severe stenosis rates were 0.3% for ADOII and 2.2% for AVPII. The Piccolo device was associated with a lower tendency for LPA stenosis.

### Modified Blalock-Taussig shunts (MBTs) vs. ductal stenting (DS)

Mini et al. conducted a retrospective comparison of MBTs and DS in 127 patients with duct-dependent pulmonary circulation. Both techniques achieved similar primary outcomes—progression to planned surgery without complications—with rates of 77.5% for DS and 75% for MBTs. However, MBTs were associated with higher rates of hospital deaths, ECMO use, and major complications (ORs: 5, 0.8, and 4, respectively; *P* < 0.05). In patients with a ductal curvature index (DCI) > 0.45, MBTs yielded better outcomes (64%) compared to DS (20%). Conversely, DS was more effective in patients with DCI <0.45, pulmonary atresia, and intact septum with right ventricle-dependent coronary circulation, achieving successful outcomes in 74.1% of cases, while MBTs had poorer outcomes. MBTs are beneficial for patients with tortuous ducts, whereas DS is preferable in other scenarios.

## Conclusions and future directions

In this editorial, we have reviewed 21 manuscripts that highlight a range of innovative approaches and technologies in pediatric cardiology. These studies demonstrate significant advancements in device-based interventions and imaging techniques, but they also reveal ongoing challenges. From AMS and holography-guided procedures to novel device closures and patient-specific modeling, the field is making progress in enhancing outcomes and reducing complications in CHD management. However, critical issues remain. AMS, despite their potential, still face limitations in material design and long-term performance. Similarly, advanced imaging techniques like 3D holography and multimodality image fusion require further refinement to maximize clinical impact. The variability in outcomes with different devices and techniques, such as in pmVSD closures, underscores the need for personalized approaches based on patient-specific factors.

Looking ahead, the future of pediatric cardiology will depend on optimizing device materials and design, while incorporating technologies like 3D printing, holography-guided procedures, and biocompatible materials that adapt to the growing pediatric heart. Enhanced imaging must evolve further, integrating real-time data for improved procedural accuracy. Larger, multi-center studies are crucial to understanding long-term outcomes and refining clinical guidelines, particularly regarding the impact of these innovations on children's growth, development, and quality of life. Collaboration among engineers, clinicians, and researchers will be key to overcoming current limitations and driving the next wave of innovations. Developing tailored interventions for each patient's unique anatomy and physiology will be essential, ensuring that modern technologies translate into meaningful improvements in clinical practice and ultimately enhance patient care in pediatric cardiology.

